# Supplementary material for: Qualitative evaluation of rapid implementation of remote blood pressure self-monitoring in pregnancy during Covid-19
Source: PLoS One. 2023 Mar 2;18(3):e0278156. doi: 10.1371/journal.pone.0278156 (PMC9980805; doi:10.1371/journal.pone.0278156)
Supplement: S1 Dataset — (DOCX) [file pone.0278156.s001.docx]

**Minimal Data Set**

| **Themes** | **Selected quotes from the participants** |
| --- | --- |
| **Clinical Outcomes** | I certainly got slightly lower readings at home than I ever got in a hospital environment, I think I definitely got some white coat kind of hypertension going on. (S11)  the first one at home was high, I knew I could sit for five minutes and they weren’t going to make a decision in it, I could just repeat it in five minutes. (S11)  Yeah I think I probably started on treatment a bit quicker this time cause I had started on the home monitoring, rather than the kind of fortnightly reviewed by the midwife. (S11)  I suppose if we’re seeing a subtle change with blood pressure in the early part of pregnancy, it’s whether you get treatment on board sooner rather than later which is also good, so I think that’s a benefit. (S17)  Well, I'd say for the likes of the woman with the white coat hypertension, so I know it's going to be higher for her when she's in there, but when she's at home I know her BP is fine, so it's removing that sort of worry of 'has she really got preeclampsia or essential hypertension or anything like that' because we know that is it white coat hypertension because she's totally fine when she's at home. (S21)  it's also saving girls having to come in for interventions that they might not otherwise need. Ye, interventions, we tend to find things happen with girls because if I'm going on and I'm looking at someone's blood pressure on a computer screen, and I'm inputting the blood pressure and urine straight into their notes, they're not coming in, you're not being side tracked by anything else that they're telling you, you're purely dealing with the task at hand (S23)  now that I’m used to doing it at home, it's quite reassuring that that's my actual blood pressure, but when I go to see her, as lovely as she is, I can't control how I feel so my blood pressure's raised, you know, immediately just from being around her (S27)   I think it's probably much calmer as well, doing it in your own house rather than driving to a midwife. It's probably more accurate (S29)  If I feel a bit off I like to check it. (S29)  it also let me see the difference between my blood pressure at home and my blood pressure when I got to the hospital. Obviously travelling to the hospital, and the fact that there was a pandemic going on, my blood pressure was always slightly higher at those appointments. But I could say to them, 'well I actually checked my blood pressure last night, or this morning, and this is what my blood pressure was at.' So they did recognise that going to the appointment was causing my blood pressure to rise as well. (S32)  it probably helped keep my blood pressure more in range because I I wasn't sitting in between appointments panicking, wondering if my blood pressure was going up, wondering if, if I was gonna have preeclampsia again. (S32)  it's been useful as well for some of our ladies who have white coat hypertension, for whom you know there's not, you suspect there's not an underlying hypertensive significant disorder, so, for these ladies, even actually just getting them to do flo for a week, so they're not on it long term, but even just getting them to set it up for a short term, almost like an evaluation tool, and then you find either, actually they do have genuine hypertension, so there's more to it than their anxiety about attending our midwife or medical centres, or it's entirely normal so it's reassuring for their perspective and from our perspective that there's probably nothing going on. (S35)  it's kinda useful in three ways: it's useful for the high risk ladies who have existing hypertension or high risk of developing it, so it's a good adjunct to care [for those with diabetes or gestational diabetes], it's a good assessment tool as well [for women with potential white coat syndrome], and it's helped improve or minimise face to face contact but in a safe way. (S35)  [The concern with putting women on medication early/easily is] probably the side effects for the women, you know, being on medication they're maybe not really needing, when it's borderline blood pressure. The medication is not always great for someone, and it makes them feel a bit sick with headaches and things. (S44)  more women are being commenced on medication for high blood pressure. (S55)  It made me think about my blood pressure properly, you know, usually the midwife comes and she'll do whatever, you know, so I think when it's me that's doing it you take a bit more responsibility for it I suppose (S25)  what I'm concerned about it over medicalising somebody that doesn't need it (S50)  Yeah, so I think ultimately got the same treatment but probably with less visits and it probably highlighted things a bit quicker than waiting for reviews (S11) |
| **Service Outcomes** | I certainly think from the point of view of booking we would very rarely have seen women in the first half of pregnancy from the day bed point of view (S17)  I think being able to identify those higher risk women or women who are shielding for example at booking, equally moving forward those at risk of hypertensive problems, so ladies who’s body mass is not of a healthy weight, women with multiple pregnancy diabetes, we can obviously recruit them earlier on in pregnancy and I suppose again that’s been a change for the team liaising with them because we didn’t, that was usually being led by the community midwives (S17)  I think the girls in day bed will have felt that their workload has increased, particularly with the unscheduled calls (S17)  Checking Florence is a 5 minute job to check it, it's not sort or giving me any extra work at all because I'm just quickly logging on and choosing  day when I've got that time to have a look. I've never had any abnormal results (S21)  Some people would say it is more work, more women being medicated, that’s the feedback that I'm getting (S05)  it’s increased the numbers of phone calls to the unit and this is time consuming(S05)  I would hope that the appointment time is shorter in the sense that what would have been a face to face would involve a longer appointment and we would do some other routine checks which probably are not really required. (S13)  So the care pathway in terms of frequency of appointments it would be less frequent I would say (S13)  I think that has led to a little bit of duplication of work because the assessment unit is also doing telephone follow ups for the women while the women are still having their regular midwife checks at the community midwives, so I think if it was more community based I would suspect that we would see workload to the day assessment unit would go down(S13)  it saved time for me and time for them having to come to clinic to have their BP and their urine checked. (S15)  So we have one main desk with one phone and it’s constantly going with either midwives referring people for the home blood pressure monitoring to be setup on it, or that they're seeing someone who's already on the monitoring and their blood pressure is high, or women phoning themselves; phone calls definitely and then, yeah, in that respect bringing people in has kind of increased in the last couple of months definitely (S16)  it’s very time consuming having to do the phone calls(S16)  I think it’s really, really individual cause some people on treatment will still hit the kind of 130s on 90s, 140s on 80s and still the consultants are happy for them to go home with kind of follow up, but I think it really depends on the clinical situation but I do think it has added to our workload as well that kind of treatment level of having to bring people in, get them started on treatment and then follow that up.  I think the 135 on 85 is kind of a blood pressure to get flagged up, I don’t know if we feel that’s a bit extreme because it’s quite a normal blood pressure in a lot of pregnant women, especially towards the end, will kind of hit that mark anyway, so we have been getting a lot more phone calls about that (S16)  it's a lot easier for me to do it at home so I think it's a really good idea. Particularly now with COVID and everything. (S20)  I don't have to see people for appointments that would just be for a quick BP check. They can just do it at home and that gives me more time to spend with patients that perhaps are needing that bit of extra time (S21)  Ye I mean it takes a lot of time, you know, like, twice a week, one midwife is taking out to do it, you know, to show new women how to do it, to do the phone calls in the afternoon, em, it's taken a lot of our time and initially it was sold to us that it was gonna be, it was gonna stop so many women coming in, but in my opinion, that doesn't seem to be the case, it feels like more work than less (S44)  I think it certainly increased it [the workload] at the start. I think we're definitely seeing people now from setting up the monitoring and it can increase it at the beginning, because a lot of people we would never have seen at that point becuse they would have, if they were essential hypertension, for example, they would have seen their GP and they would have been started on treatment there and we never would have really met these people, but now we're seeing them, and sometimes they get here and they aren't on treatment already, em, we want to maybe start them [on treatment] before we start the monitoring because we know that, again, as I say, they'll be on the phone within the week needing treatment. But these women are women we wouldn't have seen in the past (S52)  As far as I am concerned at the hospital then I find that I could potentially follow them up more with a telephone consultation as opposed to a face to face appointment if they don’t need any other checks at that time (S13)  in terms of frequency of appointments it would be less frequent I would say (S13)  I think that [new guidelines regarding abnormal BP parameters) caused a few more unscheduled calls to day bed, which I think continues (S16)  there's a reduction in workload in one aspect, but just maybe an increase in computer work, which I guess if you're not very technically minded, can seem like a little bit scary (S35)  although we've reduced some face-to-face blood pressure assessments, which we definitely will have, we've increased that workload at the other side (S35)  so nobody's mentioned it [an increased workload due to home monitoring] to me that it has been a concern...but then again these are very small numbers (S42)  we've had about six girls on the home blood pressure monitoring and we have set them up (S9)  Twice a week, one midwife's practically all day doing the phone calls and also, not just the phone calls, but check Florence, check the blood pressures before they phone them and check how they've been doing, so that takes a lot of time, plus we have slots for 3 or 4 women to come in to get shown how to do it (S44)  they've [women]  already been highlighted because they've come down, but the ones early on [in pregnancy] with essential hypertension, when we were showing them how to use it, but their blood pressure was always gonna be high because they've got essential hypertension and then it got confusing and then I would say 'well, what will I do?' to one of the doctors, and they'd say, 'well, you can't ignore it, you have to do a profile.', so we'd do all our monitoring and stuff. And they're only 14 weeks. So, I've had a few of them and it just takes ages and it's, to me, it felt like a waste of time. So it's not been great (S44)  we're fortunate here to have smaller numbers so I feel like I have got the capacity (S50)  people that were sort of borderline treatment level, but weren't quite treatment level, we knew they were gonna be a lot of work because they kept phoning back because as per Florence, they were told to. So there was a lot of work (S52)  I think maybe the communication between the nurses in the day assessment unit and my community midwife, I don't think the community midwife really knows what's going on, or why I've gone on. (S22)  everybody’s working flat out, with the current stresses on services and reductions in staff due to covid (S35)  When you've got small numbers [of patients coming through] and dispersed teams [embedding new initiatives is challenging] (S41)  There's always an individualised plan for that person because sometimes readings they can be high but they started off high, which is maybe less worrying than someone who started off quite low and went high (S49) |
| **Psychosocial outcomes** | it saved time for me and time for them having to come to clinic to have their BP and their urine checked (S15)  I think they're used to getting to know their ladies and having that kind of relationship, so, I suppose it’s a different way of caring for these women (S16)  I suppose it’s just a different way of delivering the service and keeping women safe from a hypertension point of view (S17)  I think it’s definitely adjusted for them.  I mean it’s good in a sense they don’t have to come out their house to come and see us, but in the same sense you can't, you know, that blood pressure might be normal but they might have symptoms, you know, like of a headache, of dizziness, of vomiting or swelling, that’s one of the big ones is swelling in regards to one of the conditions for pre-eclampsia, it’s one of the symptoms of it, and I guess that when they're inputting their blood pressure it might be normal but actually they’ve got increased swelling and a headache and dizziness. So I feel like it is good in monitoring that specific part but it’s not good in the sense that normally these girls would be coming in for us to see and we could visually see them (S08)  the fact that you're able to take it home with you meant that I literally would’ve been in hospital every single day, whereas I was able to go into hospital maybe one, two or three times a week, but also providing my readings by text message as well or I could provide them over the phone, so that was a massive help otherwise I would’ve been in hospital every day and that’s not what you want (S10)  my readings weren’t exactly great half the time so that would cause anxiety, but the fact that I could read them, know them and then phone in with them and go from there, that in itself was better rather than me having to stress about going into the hospital and stuff and then being like ‘oh my god my blood pressure’ or whatever (S10)  I had the same problem in my previous pregnancy without home monitoring, I essentially used my own home monitor but spent more time going up to day assessment unit in the first pregnancy, so no it was, yeah easy enough to do the actual reading and then get advice (S11)  Ye I thought it was a great idea really, I had high blood pressure in my last pregnancy as well, and I suppose the midwife had to come out more often, just to keep an eye on it. So the fact that I could do it at home, as well as the midwife coming to do it, I thought that was good. (S25)  this home monitoring system meant I didn't need to be out of work once or twice a week to have my blood pressure taken (S27)  midwives is about a 20 minute drive away, sometimes a bit longer, so it saves all that time as well, having to organise babysitters and stuff, so that takes less stress (S30)  It was brilliant to be honest. I didn't have to go up and down to the hospital. (S36)  I found it very reassuring in the sense that it was being monitored.(S11)  Yeah I feel like I'm getting on well with it and I find it quite reassuring and like I said to you after I have the baby I'm going to get one of these monitors, I've found it quite a positive experience and I think it is quite good (S12)  After my experience, I'd just prefer a professional to do it rather than just being in the house cos when you go to your midwife appointment as well, sometimes you're waiting 10-15 minutes and you're getting that time to chill and then you are getting the proper professional reading done (S24)  It just feels like, if you can do it at home, it makes you think a bit more about your own health, which I think is a good thing (S25)  I think the full idea of having those available for a patient to be able to monitor their own blood pressure at home is an amazing idea. I wish they'd had it previously with my other pregnancies (S31)  because of what was going on with covid and appointments being missed and cancelled last minute, I did feel that it was just 'here, have a machine do it yourself.', kind of thing (S43)  I've certainly found myself taking it on other days as well (S14)  because you've got that monitor there I've had to sort of train myself in the past few weeks of not constantly checking my blood pressure(S14)  midwives is about a 20 minute drive away, sometimes a bit longer, so it saves all that time as well, having to organise babysitters and stuff, so that takes less stress  (S29)  I think there are a few women who are doing it but don’t necessarily follow the guidance given, so I think that can be a little bit difficult because obviously this is new and there's that small anxiety, you know, that if they don’t follow, most of them are but, you know, and we have picked up when the changes in their blood pressure have been quite mildly elevated as opposed to and one who's missed significant numbers, so I think despite good counselling women sometimes don’t necessarily follow the instructions which can be a little bit worrying.(S13)  you know, there was a lady who had a plus of protein and she said ‘oh I didn’t think I had to phone till I had three pluses of protein’, you know, that kind of… obviously that’s not in any guidance or no one would have said that to her, but I think people kind of, you know, I assume they make up some of their own guidance (S13)  I think I have seen, especially when one of the readings is a little bit high and Florence says sit for five minutes and repeat the blood pressure, a lot of women don’t do that, they just do it the next day (S13)  I think if someone is not using it correctly, then there is the risk that you're perhaps missing something, but it's just making sure that the patients are aware of the reasons for using it and making sure they're being responsible for it and Florence is quite good for that, it will text the patient if they've not, em, sent the blood pressures in (S21)  I suppose the only thing would be that fact that the differences in the last couple of weeks to the hospital monitors, but I don't know, I mean. It's strange, I know obviously that the blood pressure monitor was definitely ok and working fine, em, but, I just don't understand how there was such a big difference in the last few weeks (S36)  The only risk that makes me worry is that if women is not doing it at the told frequency and we don't have any control over it (S37)  there were potential risks that maybe they wouldn't take their blood pressure, but because we were using the Florence system where we were reminding the women to take their blood pressure (S39)  women not that keen on going to hospital and if they're actually recording it themselves, I think they may, eh, sort of, eh, put in a lower reading that what it might actually be so that they don't have to go to hospital. That is one of the concerns that we actually had (S45)  as with any piece of machinery you sometimes get vastly different readings… it gave 3 quite wildly different readings so I felt like I couldn't trust what it (S49)  I think there is a potential for something to be missed there (S5)  2 of our machines that have come out with very high readings so the midwives then went out to do a manual reading and the manual reading was absolutely fine so she sent both of those monitors to medical physics to be chekced and calibrated but they should have all come out ready to roll and I know another midwife had had a similar situation as well (S50)  our fear was obviously someone would go away, not really know what they were doing, not record the, em, monitoring on the system and we would never know, unless we checked them all (S50)  And I suppose as well sometimes it’s compliance, you know, so you maybe get girls who meet the criteria but they maybe don’t comply (S8)  you can't, you know, that blood pressure might be normal but they might have symptoms, you know, like of a headache, of dizziness, of vomiting or swelling, that’s one of the big ones is swelling in regards to one of the conditions for pre-eclampsia, it’s one of the symptoms of it, and I guess that when they're inputting their blood pressure it might be normal but actually they’ve got increased swelling and a headache and dizziness. So I feel like it is good in monitoring that specific part but it’s not good in the sense that normally these girls would be coming in for us to see and we could visually see them and see maybe any changes in them (S7)  I guess in the current climate, everything's had to become online, skype, zoom, teams, and although people are getting more used to that way of working (S35)  Individual midwives are the ones that get emails from Florence to tell them that your women's not been in touch for a while…We've not always got access to our emails, you know, we're not always here (S52) |
| **Suitability of women** | it's got the benefit on 1 side if somebody is genuinely anxious about healthcare professional taking their blood pressure and they can do it in a calm and relaxed way, em, but, equally, putting that pressure on somebody to take, I think you have to know your clients and I think there are some people that might find that too much pressure (S50)  the benefit is that if you have an overly anxious person then they have that piece of mind that they have that machine there that they can press a button and it can tell them that their blood pressure is fine, equally, if you have that anxious person she could be doing it every 2 minutes, becoming more anxious that it could go up (S48)  the women, they might not, sometimes they haven't phoned when they've got protein in their urine, or they haven't phoned. So, I think it's actually really important to actually screen the women, that they're ok doing this and they've not got learning difficulties and other issues (S44)  I think because of the numbers that our healthboard look after, there's very few women that fit the profile of the women in the clinical criteria. We've also included then and extra 4th tier of women who are particularly remote rural locations, em so that is starting to happen (S50)  people that were sort of boardline treatment level, but weren't quite treatment level, we knew they were gonna be a lot of work because they kept phoning back because as per florence, they were told to. So there was a lot of work (S52) |
| **Support for women** | Yeah it was good.  It was easy to use, the introduction to it was good and I got quite regular phone advice when I needed it. (S11)  It [Florence] will remind them, and it will email us if the patient's not done it and then we can get in touch with the patient. So, there's like a fall back there, in case your patients are missed (S21)  it's also making sure that the women understand how to use things, how to record things, and how to action things so that there's absolutely no risk of someone sitting there with a result that you would want acted on for example and it's not connected in (S42)  everyone will tell you they understand what you're saying to them, em, but, on the back of that we wanted to check that they were understanding and that they were doing it properly (S50)  for me I always like to just check in and make sure that everything else is okay, cause I suppose it is only monitoring a small fraction of what we are concerned about, it monitors the kinda physical attributes such as blood pressure and urinalysis but it doesn’t monitor other symptoms (S7) |
| **Staff buy-in** | it hasn't been as popular as much as I thought it was going to be implemented and I think it may be down to we've been saturating the midwives with so much (S39)  although the midwives have embraced it to a certain extent, if you're not getting it requested from the obstetrician to put it on then there are maybe some midwives who think 'well I don't need it because I'm not being asked about it from the health prof who’s leading this women’s care'(S39)  the biggest barrier was engagement from the obstetricians, believe it or not, cos I felt as if if they'd have been more engaged at the very beginning, em, then they would have been instructing the midwives (S39)  because of our geography, so we obviously have to get the buy in from a consultant [obstetrician] who's not in [our health board] (S41)  we know that every time we introduce something new with midwives as much as anything, it's another barrier very often, because it's something else to take on and something else to learn (S41)  they cannot set it up until they get a green light from management to do that  (S5)  Poor consultant that has been our contact all the way through because we've had to get in touch with the consultant because really not a lot, there's not much buy in, there wasn't a lot of buy in from a lot of the other doctors in the hospital or they weren't aware of it like they should be (S52) |
| **Staff time and capacity** | It was good but there was a lot of things going on. We've got a lot of pressures. There's been manditory training, national education programmes have come out, have we saturated out midwives brains, and maybe that's why, although I thought the training was really good, em, I thought we had really good support and I think it's a reallty good thing for women, and there's really good national criteria for who we should be putting onto it, it hasn't been as popular as much as I thought it was going to be implemented and I think it may be down to we've been saturating the midwives with so much (S39)  initially we thought it would be on one morning we have both face to face and telephone consultations but obviously that meant taking more staff out of the current running of the day assessment unit (S10)  it’s very time consuming having to do the phone calls (S16)  there's more unscheduled calls which the girls [midwives] are having to deal with which have been a challenge for them I think (S17)  It was good but there was a lot of things going on. We've got a lot of pressures. There's been mandatory training, national education programmes have come out, have we saturated our midwives brains (S37)  there's really good national criteria for who we should be putting onto it (S37)  we had also just rolled out BadgerNet, so between Badgernet, covid, eh, Near Me, everything just seemed to come at one time and it was quite a lot for, em, well we had guidance on covid, it was quite a lot for the midwives themselves to take in (S45)  I think the time factor with everything else that was actually going on, that was a big challenge, the time factor, and again, due to staffing levels and things as well (S45)  We didn't have the staff to cope with it (S52) |
| **Training and clinical guidance** | I don’t feel like we do have that clear guidance (S16)  there's a slightly grey area between 135/85 to 139/89 (S17)  I don't think we've had enough support at the beginning of it and a lot of the time, we're not really sure what we're doing…I think if they'd had all the guidelines and the exact flowcharts of what to do when A, B, or C happens at the beginning, it would have been much smoother (S44)  There could have been a lot more work done before it started about how to implement it. Pathways, guidelines, you know, education sessions, we've never had any education sessions, about it (S44)  I think it would have been really good if there had been more discussion with team leads before this had actually been rolled out (S45)  there's not clear guidance on how often their supposed to do it, when you're supposed to do it, how often  you're still supposed to see your midwife in between and if you're unwell, well unwell is different for different people (S49)  I think there's probably a lack of guidance written down as to what to do when it deviates from normal (S49)  Well, at the start, we weren't 100% clear what we were supposed to be doing if I'm truly honest, because we had no set protocol in place when we took it on (S50)  I mean even at the start we weren't sure if we, we knew we had to phone them back, but we weren't sure if it was one week, we weren't sure if it was 2 weeks, who were we phoning back, should we be phoning everybody back, you know, it just wasn't clear for anyone, who we should be phoning and what we should be doing (S50) |
| **Infrastructure and equipment** | some phone providers that have actually blocked the texts, so even although it’s a free service it’s been blocked (S5)  The setup itself, you know, we have three computers really but one of the computers is in an office that gets used by other people as well, so it can be difficult finding somewhere quiet and confidential to do the phone calls (S16)  all of them actually came in a small size and a large cuff I think would have been helpful because there have been some women that have been unsuitable because the cuffs haven’t fit them properly and it’s given them too high a reading cause it’s been too small (S16)  some phone networks don’t actually allow the texts for Florence to come through.  It was either… it was two words, it was either Talk Talk or Giff Gaff(S16)  I suppose the only thing would be that fact that the differences in the last couple of weeks to the hospital monitors, but I don't know, I mean. It's strange, I know obviously that the blood pressure monitor was definitely ok and working fine, em, but, I just don't understand how there was such a big difference in the last few weeks (S36)  The [larger] cuffs didn't come until about 6 weeks after [the monitors]. Women that we probably would have put onto BP monitoring at home, we couldn't because we didn't have the larger cuffs at that time (S45)  there's a huge barrier because of lack of mobile phone signals in various areas (S45)  IT has been our biggest barrier here (S45) |
